# Identification and Characterization of MicroRNAs in the Liver of Blunt Snout Bream (*Megalobrama amblycephala*) Infected by *Aeromonas hydrophila*

**DOI:** 10.3390/ijms17121972

**Published:** 2016-11-25

**Authors:** Lei Cui, Hongtao Hu, Wei Wei, Weimin Wang, Hong Liu

**Affiliations:** 1 College of Fisheries, Key Lab of Freshwater Animal Breeding, Ministry of Agriculture, Huazhong Agricultural University, Wuhan 430070, China; cuilei.1987@webmail.hzau.edu.cn (L.C.); 18314787156@163.com (W.We.); wangwm@mail.hzau.edu.cn (W.Wa.); 2 Center for Bio-Pesticide Research, Hubei Academy of Agricultural Sciences, Wuhan 430064, China; hongtaohu@hbaas.com; 3 Freshwater Aquaculture Collaborative Innovation Center of Hubei Province, Wuhan 430070, China

**Keywords:** miRNAs, *Megalobrama amblycephala*, *Aeromonas hydrophila*, deep sequencing, immune response

## Abstract

MicroRNAs (miRNAs) are small RNA molecules that play key roles in regulation of various biological processes. In order to better understand the biological significance of miRNAs in the context of *Aeromonas hydrophila* infection in *Megalobrama amblycephala*, small RNA libraries obtained from fish liver at 0 (non-infection), 4, and 24 h post infection (poi) were sequenced using Illumina deep sequencing technology. A total of 11,244,207, 9,212,958, and 7,939,157 clean reads were obtained from these three RNA libraries, respectively. Bioinformatics analysis identified 171 conserved miRNAs and 62 putative novel miRNAs. The existence of ten randomly selected novel miRNAs was validated by RT-PCR. Pairwise comparison suggested that 61 and 44 miRNAs were differentially expressed at 4 and 24 h poi, respectively. Furthermore, the expression profiles of nine randomly selected miRNAs were validated by qRT-PCR. MicroRNA target prediction, gene ontology (GO) annotation, and Kyoto Encylopedia of Genes and Genomes (KEGG) analysis indicated that a variety of biological pathways could be affected by *A. hydrophila* infection. Additionally, transferrin (*TF*) and transferrin receptor (*TFR*) genes were confirmed to be direct targets of miR-375. These results will expand our knowledge of the role of miRNAs in the immune response of *M. amblycephala* to *A. hydrophila* infection, and facilitate the development of effective strategies against *A. hydrophila* infection in *M. amblycephala*.

## 1. Introduction

MicroRNAs (miRNAs), typically ~22 nucleotides (nt) in length, are small non-coding RNAs, and produced from longer, hairpin-shaped precursors (pre-miRNAs) by two RNase III proteins, Dicer and Drosha with the assistance of DiGeorge syndrome critical region 8 (DGCR8) [1,2]. Mature miRNAs are loaded onto Argonaute proteins (AGOs) to form RNA-induced silencing complex (RISC) in which miRNAs function as a guide by base pairing with target mRNAs, leading to cleavage of target mRNAs or translation repression. Since the discovery of the first miRNA, *lin-4*, in *Caenorhabditis elegans* in 1993 [3], thousands of miRNAs have been identified in animals, plants and viruses. Recently, with the rapid development of high-throughput sequencing technology, a large number of miRNAs have been discovered in fish, such as rainbow trout (*Oncorhynchus mykiss*), channel catfish (*Ictalurus punctatus*), grouper (*Epinephelus coioides*), grass carp (*Ctenopharyngodon idella*), common carp (*Cyprinus carpio*), and blunt snout bream (*Megalobrama amblycephala*) [4,5,6,7,8,9]. Most miRNAs are conserved between species, and their expression exhibits time-dependent and tissue-specific characteristics [10,11,12].

A multitude of studies demonstrate that miRNAs play crucial roles in the regulation of diverse biological processes, including cell differentiation, apoptosis, and immune defense [13,14]. In addition, increasing studies have revealed that miRNAs are crucial for host responses to pathogen infection in eukaryotes. In vertebrates, a subset of miRNAs, including miR-146, miR-155, miR-21 and let-7 family members, have been found to contribute to immune responses during bacterial infections [15,16,17]. For instance, let-7f regulates immune responses to *Mycobacterium tuberculosis* infection by targeting A20, a feedback inhibitor of the NF-κB pathway in mammals [17]. In zebrafish embryos, with *Salmonella typhimurium* infection, miR-146 was induced in a myeloid differentiation factor 88 (*MyD88*) and tumor necrosis factor receptor-associated factor 6 (*TRAF6*)-dependent manner, and knockdown of miR-146 resulted in the increase of apolipoprotein genes during inflammation [16]. Recent studies demonstrate that miRNAs also play pivotal roles in the metabolism of iron, an essential element for the growth and survival of most organisms including fish and bacteria, and a key regulator of host-pathogen interactions [18,19,20,21].

Blunt snout bream, a herbivorous and endemic species in China, belongs to *Megalobrama*, Cyprinidae, Cypriniformes, Actinopterygii, and is naturally distributed in the middle and lower reaches of the Yangtze River [22]. Because of its delicacy, *M. amblycephala* has been widely cultured as a major aquaculture species in the Chinese freshwater polyculture system. However, recent bacterial septicemia from *Aeromonas hydrophila* infection has caused great economic losses in the *M. amblycephala* aquaculture industry. In order to develop effective strategies for disease management, elucidation of the processes involved in the interaction between *M. amblycephala* and *A. hydrophila* is essential. Studies concerning the response of miRNAs to bacterial infections have confirmed miRNAs as key players in the host innate and adaptive immune responses [23], suggesting that identification of immune-related miRNAs might facilitate the understanding of their pathological functions and the disease defense mechanisms in *M. amblycephala*. To date, miRNAs have been found to be involved in growth, diet-induced hepatic steatosis, intermuscular bone development and lipopolysaccharide (LPS) stimulation in *M. amblycephala* [8,24,25,26]. However, the involvement of *M. amblycephala* miRNAs in *A. hydrophila* infection has not been elucidated.

In the present study, we identified conserved and putative novel miRNAs in *M. amblycephala* liver at different time points post-infection with *A. hydrophila* using Illumina deep sequencing. The expressions of nine randomly selected miRNAs were validated by quantitative real-time PCR (qRT-PCR). GO annotation and KEGG analysis showed that the putative miRNA target genes are involved in a variety of biological pathways, particularly immune responses. Additionally, miR-375 was demonstrated to target transferrin (*TF*) and transferrin receptor (*TFR*) genes. These results will provide novel insights into the mechanisms of miRNA-mediated regulation of the immune response after *A. hydrophila* infection in *M. amblycephala*, and facilitate the development of effective disease prevention measures and genetic improvement methods for *M. amblycephala*.

## 2. Results

### 2.1. Small RNA Analysis in the Three Libraries of the M. amblycephala Liver

**Figure 1:**
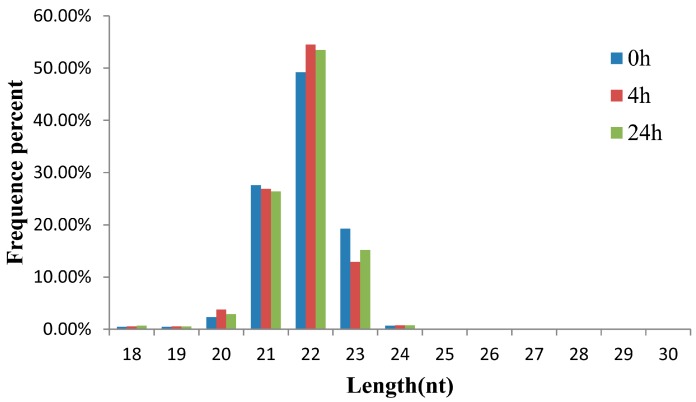
Length distribution of small RNAs in the liver of *M. amblycephala* at various time points post-infection with *A. hydrophila*.

**Figure 2:**
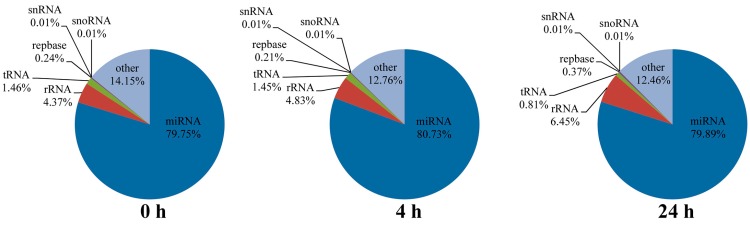
Annotation of small RNA libraries generated from the liver of *M. amblycephala* at various time points post-infection with *A. hydrophila*. The charts show the percentage of small RNA to the total number of clean reads.

The small RNA transcriptome of *A. hydrophila*-infected *M. amblycephala* was analyzed in the liver because the organ plays important roles in immune responses in both mammals and fish, particularly in fish responses to *A. hydrophila* infection [26,27,28,29,30]. Previous studies in our lab indicated that some immune-related genes are activated at an early stage of *A. hydrophila* infection in *M. amblycephala* [31,32,33,34,35]. Therefore, three small RNA libraries were analyzed from *M. amblycephala* liver at 0 (non-infection), 4, and 24 h post infection (poi) using the Illumina Hiseq 2000 sequencing platform. A total of 13,916,974, 11,975,199 and 10,468,772 raw reads were collected from the three libraries, respectively. After removing the sequencing adaptor and low quality reads, 11,244,207, 9,212,958 and 7,939,157 high quality clean reads were retained for mapping analysis (Table S1-1), and the length distribution of clean reads was summarized to assess the sequencing quality. As shown in Figure 1, clean reads in the three libraries shared a very similar length distribution, dominated by 22 nt small RNAs, followed by 21 and 23 nt, which were consistent with the typical sizes of small RNAs in other fish species, such as channel catfish [5], grass carp [9] and common carp [7]. In total, 863,710, 589,635 and 547,454 unique sequences were obtained from the three libraries, respectively. Most of the unique sequences were specific to one library, and only a few were included in at least two libraries. Collectively, common sequences accounted for more than 80% of the total sequences (Table S1-2). After comparing the clean sequences with the Rfam, GenBank and Repbase databases, the rRNA, tRNA, snRNA, snoRNA and repeats were annotated and removed. Finally, 8,967,663, 7,437,425 and 6,342,311 reads of the three libraries were retained for the subsequent miRNA analysis (Figure 2 and Table S1-3).

### 2.2. Identification of Conserved and Novel miRNAs in the M. amblycephala Liver

To identify the conserved miRNAs in the *M. amblycephala* liver, we compared the above collected data to the known mature miRNAs in the miRbase 21.0 (http://www.mirbase.org/). A total of 171 conserved miRNAs belonging to 84 families were identified (Table S2). Among them, 168 were identified in all the three libraries, one (miR-153b) was identified in the 0 and 24 h libraries and two (miR-2187-5p and miR-459-5p) were identified in the 0 and 4 h libraries. The read counts of the conserved miRNAs ranged from 1 to 4,185,500, indicating their wide expression range. miR-122, miR-22a, miR-100, miR-192 and miR-21 were five of the most abundant miRNAs in all the three libraries. The top 10 most abundant miRNAs are shown in Table 1, accounting for 85.86%, 80.62%, and 82.25% of all the conserved miRNAs read counts in the 0, 4 and 24 h libraries, respectively.

**Figure 3:**
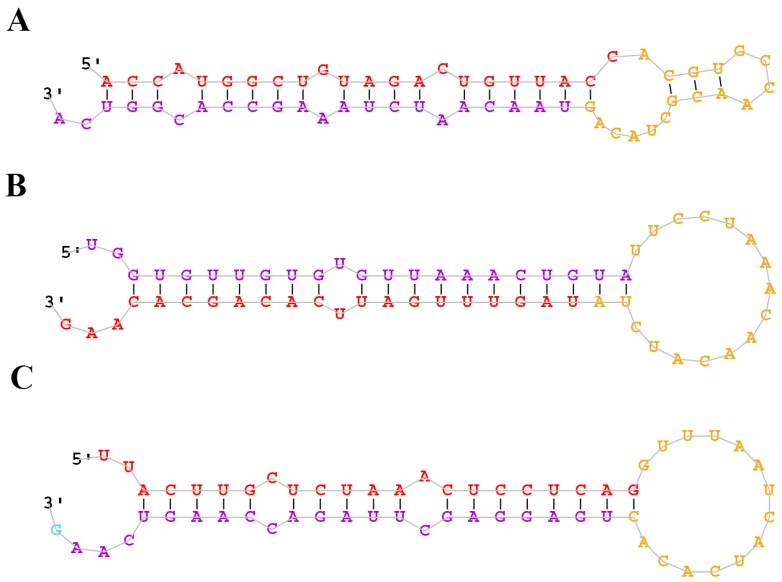
Predicted stem-loop secondary structures of selected *M. amblycephala* putative novel miRNAs. Dominant forms of the mature miRNAs are highlighted in red. (**A**) miR-novel 3; (**B**) miR-novel 55; and (**C**) miR-novel 62.

**Figure 4:**
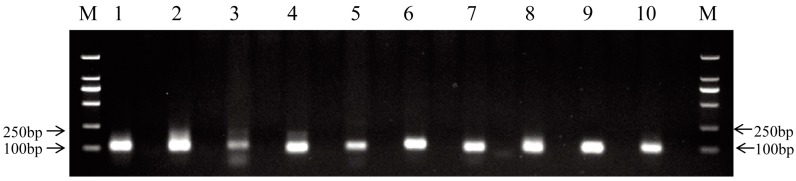
Validation of novel miRNAs by RT-PCR. **M**: DL2000 marker; **Lanes 1**–**10**: novel miRNAs (miR-novel 3, miR-novel 7, miR-novel 13, miR-novel 20, miR-novel 24, miR-novel 28, miR-novel 33, miR-novel 34, miR-novel 41 and miR-novel 54). For each miRNA, the right lane is the negative control.

Due to the unavailability of the *M. amblycephala* genome sequence, the sequences that did not match the known miRNA precursors were mapped to the zebrafish genome and analyzed by miRDeep2 (mapper. Pl config_miRDeep; parameters: -e, -d, -h, -i,-j, -l, 18, -m and -p) [36] to identify potential novel miRNAs based on secondary structure, the Dicer enzyme cleavage site and the minimum free energy. The miRNAs with miRDeep2 score ≥0 were considered as putative novel miRNAs, and a total of 62 putative novel miRNAs were identified (Table S3). According to the results of miRDeep2, the putative novel miRNAs were randomly named in the present study. The predicted secondary structures of the novel miRNAs are shown in Figure 3 and Figure S1. Of the 62 novel miRNAs, miRNA-novel 60 was identified only in the 0 h library. Intriguingly, the expression level of most putative novel miRNAs was much lower than that of conserved miRNAs in the three libraries. To validate the bioinformatic predictions of novel miRNAs, RT-PCR was used to test ten randomly selected novel miRNAs from the 0 h library. As shown in Figure 4, the sizes of RT-PCR products of novel miRNAs were around 100 bp. In contrast, no product was detected in the negative controls with the templates replaced by H_2_O. The results indicated the existence of these novel miRNAs in the liver of *M. amblycephala*. Sequencing of the PCR amplicons will further verify the identities of these putative novel miRNAs.

### 2.3. Differentially Expressed miRNAs

**Figure 5:**
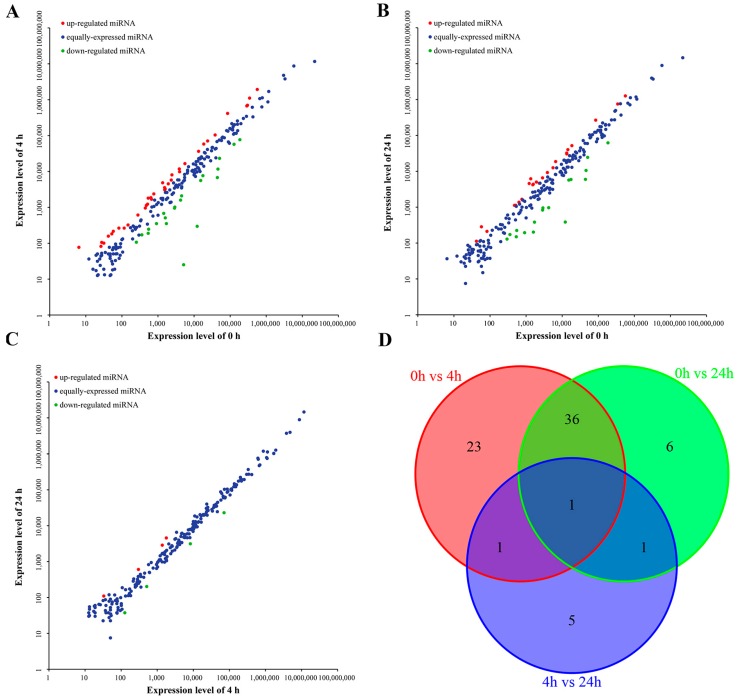
Preliminary evaluation of the differential expression of miRNAs of the three libraries in the *M. amblycephala* liver. (**A**–**C**) Scatter plot map of miRNAs expression levels in the 0, 4 and 24 h libraries. Each dot represents an individual miRNA. Red dots show apparently up-regulated miRNAs and green dots show apparently down-regulated miRNAs; (**D**) The Venn diagram shows the distribution of differentially expressed miRNAs of the three libraries. The overlapping section represents the number of co-expressed differentially expressed miRNAs.

To identify miRNAs involved in immune function in the *M. amblycephala* liver after infection with *A. hydrophila*, the expression profiles of miRNAs from the three libraries were compared to uncover their differential expression. Each time point was represented by a single Illumina library, differences could therefore be due to technical variations. Still, a comparison was performed as a preliminary evaluation of differential expression. The results were shown by scatter plot map (Figure 5A–C). A total of 73 miRNAs were identified to potentially exhibit differential expression in at least one pair of libraries compared (Table S4). Of the 73 miRNAs that potentially vary between different time points, 38 and 24 miRNAs appeared up-regulated, while 23 and 20 miRNAs appeared down-regulated at 4 and 24 h poi, respectively. However, when compared to 4 h poi, only eight miRNAs were differentially expressed at 24 h poi, including four up-regulated (miR-301c, miR-novel 2, miR-novel 11 and mR-novel 56) and 4 down-regulated miRNAs (miR-10a-5p, miR-10b, miR-153c, miR-499). Additionally, the distribution of differentially expressed miRNAs in the three libraries is shown in Figure 5D, with only one miRNA (miR-499) differentially expressed in all three pairwise comparisons. Collectively, 61 of the 73 (83.56%) miRNAs were up- or down-regulated at 4 h poi, implying that most miRNAs were activated in the liver of *M. amblycephala* at an early stage after *A. hydrophila* infection. Moreover, several differentially expressed miRNAs had relatively high expression levels (RPKM > 10,000) in all the three libraries, such as miR-152, miR-148, miR-101a, miR-141, miR-200c and miR-216b.

### 2.4. Validation of miRNAs by qRT-PCR

**Figure 6:**
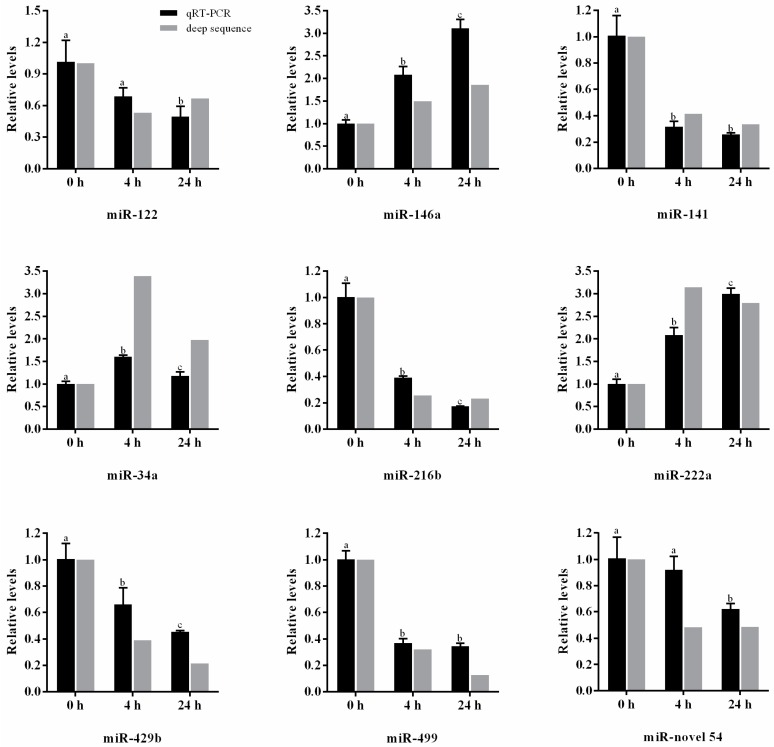
Relative expression levels of nine selected miRNAs in the three libraries according to qRT-PCR and high-throughput sequencing. The expression levels of miRNAs in the 0 h library were set to 1. Values were described as mean ± SD (*n* = 3 pools, with 7 fish per pool). Differences were determined by one-way analysis of variance (ANOVA). Different letters above pillars indicated statistical significance at the level of *p* < 0.05.

qRT-PCR was employed to validate the expression of nine selected miRNAs, which included two highly expressed conserved miRNAs (miR-122 and miR-146a), six differentially expressed conserved miRNAs (miR-141, miR-34a, miR-216b, miR-222a, miR-429b andmiR-499) and one putative novel miRNA (miR-novel 54). As shown in Figure 6, the expression profiles of these nine miRNAs were consistent with the results obtained by deep sequencing, despite a slight difference in fold changes in the three libraries.

### 2.5. Target Prediction of Differentially Expressed miRNAs and Functional Analysis

To gain insights into the functions and possible roles of the differentially expressed miRNAs, the candidate target genes were predicted by the Miranda and RNAhybrid methods and the common target genes were selected for functional analysis. A total of 113,116 putative target genes including 87,120 EST sequences of *M. amblycephala* were predicted for 233 miRNAs, and from them, 87,486 (63,086 EST sequences) target genes of the 73 differently expressed miRNAs were obtained. From the EST sequences, 87 immune-related genes were selected for further analysis (Table S5). These genes were related to interferon, interleukin, tumor necrosis factor, chemokine, toll-like receptor and complement component which play pivotal roles in resisting the bacterial invasion [37,38,39,40]. A majority of these genes were targeted by multiple miRNAs at different sites, but several of them were targeted by only one miRNA.

Furthermore, the specific functions of the differentially expressed miRNAs were identified by Gene Ontology (GO) annotation and Kyoto Encylopedia of Genes and Genomes (KEGG) pathway analyses of the target genes. Table S6 and Figure S2 present the complete GO analysis results of the predicted target genes. Interestingly, several GO terms were associated with immune response and immune system development such as toll-like receptor signaling pathway, immune system process, cytokine regulation, inflammatory response, and T and B cell proliferation and differentiation, implying the potential vital biological functions of these predicted target genes in response to *A. hydrophila* infection. Additionally, a total of 216 pathways were predicted by KEGG mapping (Table S7).

### 2.6. Effects of miR-375 on the Expression of Transferrin and Transferrin Receptor Genes

**Figure 7:**
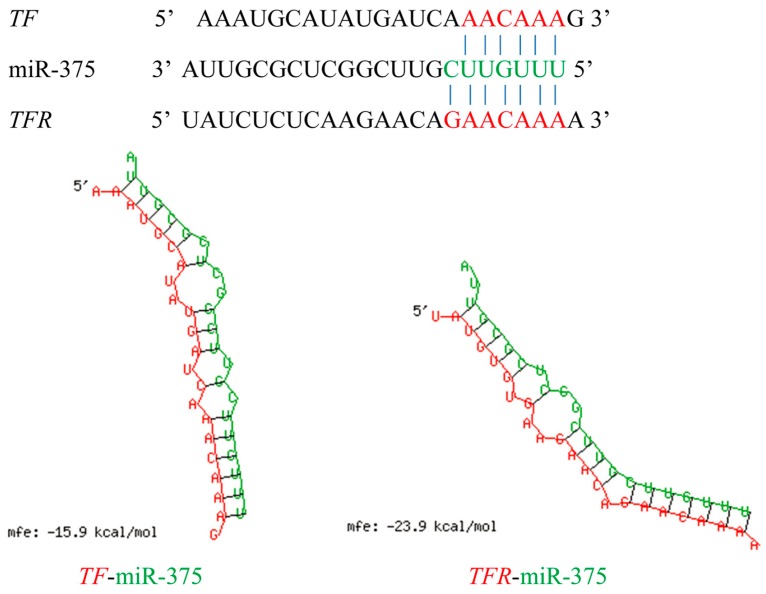
The predicted binding sites of miR-375 to transferrin (*TF*) and transferrin receptor (*TFR*) genes. The sequences of gene (*TF* and *TFR*) and miR-375 are shown in red and green, respectively.

**Figure 8:**
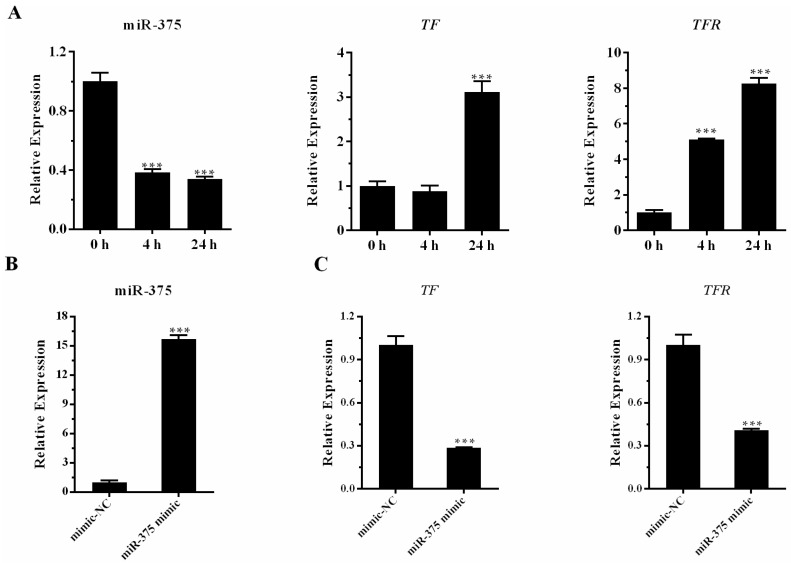
Expression analysis of miR-375, *TF* and *TFR*. (**A**) Relative expression of miR-375, *TF* and *TFR* in the three libraries; (**B**) Transfection efficiency of miR-375 mimic was detected by qRT-PCR. Values were described as mean ± SD (*n* = 3, *** *p* < 0.001); (**C**) *TF* and *TFR* mRNA expressions were detected by qRT-PCR. Values were described as mean ± SD (*n* = 3, *** *p* < 0.001).

*TF* and *TFR* genes play vital roles in iron homeostasis, which is essential for the growth and survival of fish and bacteria. Upon bacterial infection, *TF* and *TFR* can create a bacteriostatic environment by reducing the availability of iron to pathogens for proliferation [41,42]. In this study, we predicted that the differentially expressed miR-375 could bind to *TF* and *TFR* genes. The predicted binding sites of the two genes are shown in Figure 7. The relative expression profiles of miR-375, and its corresponding target genes, *TF* and *TFR*, from the three libraries were investigated. The relative expression of miR-375 was down-regulated both at 4 and 24 h poi, while its target genes, *TF* and *TFR*, were up-regulated (Figure 8A). To further confirm the effect of miR-375 on endogenous *TF* and *TFR* mRNA expressions, the miR-375 mimic and negative control (mimic-NC) were used to treat MAF cells (*M. amblycephala* fin cells). The transfection efficiency was determined by qRT-PCR (Figure 8B). The qRT-PCR results showed that the *TF* and *TFR* mRNA levels were significantly decreased by miR-375 mimic compared with the control group (Figure 8C). The above results indicated that miR-375 can negatively regulate the endogenous mRNA expressions of *TF* and *TFR*.

**Figure 9:**
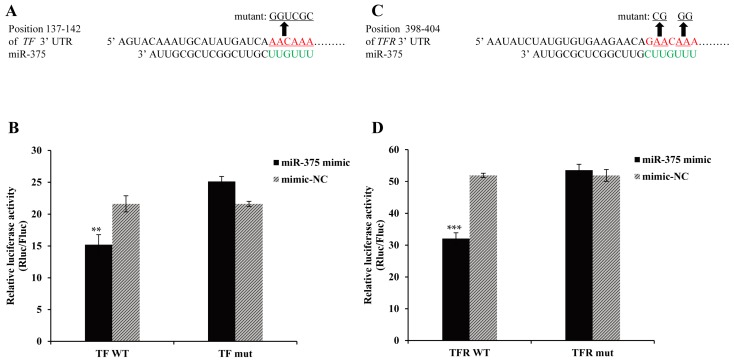
miR-375 directly targets the 3’ UTR of *TF* and *TFR* genes. (**A**,**C**) miR-375 seed sequence and its complementary sequences on 3’ UTR are shown in red and green, respectively. Mutant sites are indicated by arrows and underlined; (**B**,**D**) miR-375 mimic or mimic-NC were co-transfected with *TF* or *TFR* 3’ UTR or mutant dual-luciferase reporters into HeLa cells. Renilla luciferase activities were detected and normalized to firefly luciferase. Values were described as mean ± SD (*n* = 3, ** *p* < 0.01, *** *p* < 0.001).

To further determine whether miR-375 directly interacts with the 3’ UTR of *TF* and *TFR*, the dual-luciferase reporter constructs containing *TF* or *TFR* 3’ UTR (wild-type or mutant) (Figure 9A,C) were co-transfected with miR-375 mimic or mimic-NC. The results showed that the luciferase activity of the wild-type construct was significantly repressed by miR-375 mimic, but not that of the mutant construct (Figure 9B,D). Together, these results indicated that miR-375 modulates *TF* and *TFR* expression by directly targeting the 3’ UTR of *TF* and *TFR*.

## 3. Discussion

Since the discovery of their importance in post-transcriptional gene regulation, miRNAs have been extensively investigated in many organisms. To date, many reports have revealed that miRNAs participate in the regulation of immune responses and bacterial infection [7,14,16,43]. However, *M. amblycephala* miRNA responses during *A. hydrophila* infection have not been studied. In the present study, we investigated three small RNA libraries from the *M. amblycephala* liver after *A. hydrophila* infection at 0, 4 and 24 h poi using Illumina deep sequencing, identified a total of 171 conserved miRNAs and 62 potential novel miRNAs, and analyzed their expression profiles.

Previous studies have demonstrated that evolutionarily conserved miRNAs usually have more abundant expressions than novel miRNAs [8,43]. In this study, several evolutionarily conserved miRNAs were also found to have abundant expressions in the three libraries, such as miR-122, miR-22a, miR-192, miR-100 and miR-21 (Table 1), which is consistent with previous reports in half-smooth tongue sole (*Cynoglossus semilaevis*) and common carp [7,43]. It has been reported that miR-122, a mammalian liver-specific miRNA, was related to hepatitis C virus infection [44], and its expression was down-regulated after pathogen infection, but still remained at a high level [43,45]. Similarly, our results showed that miR-122, the most abundant miRNA in all the three libraries, was significantly down-regulated at 4 and 24 h poi (Figure 6), suggesting that miR-122 may play a critical role in host defense against pathogenic microorganisms. Meanwhile, miR-22a and miR-192 had high expression levels during metamorphosis of the Japanese flounder (*Paralichthys olivaceus*) [46]. Collectively, the abundantly expressed miRNAs identified in this study are conserved with other species, and may play an important role in regulation of fundamental biological processes and immune responses in the liver of *M. amblycephala* after *A. hydrophila* infection. Further study should focus on elucidating the precise roles of these miRNAs.

Among the differentially expressed miRNAs, miR-10a-5p was decreased at 24 h poi compared with 4 h poi. A similar expression pattern of miR-10a was observed in *Apostichopus japonicus* with skin ulceration syndrome compared to the healthy group [47]. A recent study reported that microbiota regulated the expression of IL-12/IL-23p40 through the inhibitor of miR-10a, which may contribute to the maintenance of intestinal homeostasis in mice [48]. The result indicated that miR-10a-5p is crucial for the maintenance of immune system homeostasis in *M. amblycephala*. On the other hand, miR-499 was also significantly down-regulated at both 4 and 24 h poi. The predicted target gene of miR-499, interleukin enhancer binding factor 2 (ILF2) also known as NF45, is a subunit protein of nuclear factor of activated T-cells (NFAT), which is essential for the expression of interleukin-2 [49]. This finding suggested that miR-499 may serve as a novel regulator in immune response to bacterial infection.

Several differentially expressed miRNAs have been found to be immune-related in mammals, such as miR-148/152, miR-101a, miR-141/200c, miR-146b, miR-217 and miR-375 [50,51,52,53,54,55,56]. A previous study demonstrated that the miR-148 and miR-152, two members of miR-148 family, were significantly increased in dendritic cells by TLR agonists, which inhibited the up-regulation of MHC II expression and the production of cytokine via targeting CaMKIIα, and might contribute to immune homeostasis [50]. In the present study, miR-152 was the most highly expressed conserved miRNA at both 4 h (4.89-fold) and 24 h (3.14-fold) poi, and miR-148 was also up-regulated at 4 h (3.42-fold) and 24 h (2.24-fold) poi. miR-101a, a highly up-regulated miRNA at both 4 and 24 h poi in this study, can regulate the innate immune response to LPS via its target gene MAPK phosphatase-1 in macrophage cells [51]. Therefore, we speculate that enhanced miR148/152 and miR-101a expression may regulate the expression of some immune-relevant genes to prevent excessive immune responses during bacterial infection.

On the other hand, miR-141/200c, miR-217 and miR-375 were decreased after infection at both 4 and 24 h poi. miR-141 and miR-200c, belonging to the highly conserved miR-200 family, whose members are considered as tumor suppressors [52], can promote the mesenchymal-epithelial transition, and inhibit cell invasion by suppressing the *Zeb2* and *Snail1* transcriptional repressor complexes [53]. In addition, miR-200b/c and miR-217 regulate the expression of mortalin and the quantity of MACs (complement membrane attack complex) deposited on the target cells during complement activation to resist complement membrane attack [55], and the decreased miR-375 can activate JAK2-STAT3 pathway and promote the proliferation and migration of gastric epithelial cells in response to *Helicobacter pylori* infection [56]. Meanwhile, the prediction of target genes indicated that toll-like receptor 5b (*TLR5b*), interleukin-1 receptor I (*IL1RI*), interleukin enhancer binding factor 3a (*ILF3a*) and interleukin-17 receptor B (*IL17R B*) are collectively targeted by miR-217. Innate immunity is the first line of host defense against pathogen invasion. Toll-like receptor (TLR) and cytokine signaling play pivotal roles in the innate immunity by recognizing multiple highly conserved pathogen-associated molecular patterns and activating inflammatory response [57]. Interestingly, miR-146b, a negative regulator of TLR and cytokine signaling, was repressed at 4 h poi in this study, which may up-regulated its targets, the TLR signaling intermediates TRAF6 and IRAK1, thereby enhancing the inflammatory response to *A. hydrophila* infection [54]. However, excessive or inappropriate inflammatory responses can lead to tissue injury. A previous study has reported that miR-146b was significantly down-regulated in grass carp susceptible to *A. hydrophila* in contrast to *A. hydrophila*-resistant fish [9], suggesting that the decreased miR-146b may be detrimental to the fish. Taken together, these results revealed that appropriate regulation of miR-146b expression is critically important for fish to resist infection. However, several reported immune-related miRNAs were not differentially expressed in the three libraries, including let-7 family, miR-155 and miR-192 [58,59,60], probably due to species- or tissue-specific miRNA expression [7,10].

Moreover, GO and KEGG analyses of putative targets of differentially expressed miRNAs revealed their potential involvement in host immunity, including phagosome, MAPK signaling pathway, ECM-receptor interaction, focal adhesion, cytokine-cytokine receptor interaction and toll-like receptor signaling pathway. Thus, most of the differentially expressed miRNAs might participate in the *M. amblycephala* immune response to *A. hydrophila* infection by regulating the expression of related genes in several immune-related pathways.

miRNAs have been previously demonstrated to regulate iron metabolism [61,62,63]. In the present study, miR-375 was shown to target *TF* and *TFR*, which are crucial in iron metabolism and homeostasis [64]. miR-375 was significantly down-regulated in *M. amblycephala* liver following *A. hydrophila* infection, whereas *TF* and *TFR* expression was highly increased, consistent with the inverse relationship in the expression of miRNAs and their target mRNAs [65]. *TF* and *TFR* up-regulation could lead to the endocytosis of TF-bound iron, subsequently limiting the availability of circulating iron to pathogens [41,64,66,67]. We speculate that during *A. hydrophila* infection in *M. amblycephala*, miR-375 down-regulation could be an antibacterial mechanism by allowing the expression of *TF* and *TFR* to create a bacteriostatic environment [41,42]. These results emphasize a critical role for miR-375 in the regulation of iron homeostasis during bacterial infection.

## 4. Materials and Methods

### 4.1. Fish and Bacterial Challenge

This study was performed in accordance with the guidelines of the Institutional Animal Care and Use Committees (IACUC) of Huazhong Agricultural University (HZAU), Wuhan, China (HZAUFI-2014-0019, Approval date: 27 August 2014). The specimens of blunt snout bream were collected from Tuanfeng Fish Breeding Base (Huanggang, China). The fish were acclimatized in aerated water at 28 ± 2 °C for two weeks before sampling and challenge experiment. After acclimation, healthy individuals were selected for challenge experiment with *A. hydrophila* suspension (isolated from diseased *M. amblycephala* in Dongxi Lake, Wuhan, China) using the method as described previously [64]. Briefly, fish with an average weight of 24.68 g were intraperitoneally injected with 0.1 mL suspension of *A. hydrophila* (1 × 10^6^ CFU/mL). There were 21 individuals (divided into three pools, one pool for sequencing) randomly dissected to remove the liver at each time point, 0 (non-infection), 4 and 24 h poi. The samples were immediately frozen in liquid nitrogen and then stored at −80 °C for further analysis. In order to confirm infection, *A. hydrophila* was recovered from challenged fish exhibiting typical symptoms of bacterial septicemia and confirmed by PCR amplification of the *16S r*DNA as described previously [68]. The PCR products were sequenced and BLASTed against known sequences of *A. hydrophila 16S r*DNA in NCBI.

### 4.2. RNA Extraction, Library Construction and Sequencing

Total RNAs were extracted from each of the equally mixed liver samples in the same treatment group, using TRIzol reagent (Invitrogen, Carlsbad, CA, USA) following the manufacturer’s instructions. The quality of RNAs was examined using NanoDrop 2000 (Thermo Scientific, Wilmington, DE, USA), and the integrity was checked by RNA 6000 Nano Assay using Agilent 2100 Bioanalyzer (Agilent Technologies, Palo Alto, CA, USA). Equal amounts of total RNAs from each of the three groups were used for library preparation and sequencing.

Three libraries were generated using NEBNext^®^ Multiplex Small RNA Library Prep Set for Illumina^®^ (NEB, New England Biolab, Ipswich, MA, USA). Briefly, NEB 3’ SR Adaptor was directly and specifically ligated to the 3’ end of miRNA, and after the 3’ ligation reaction, the SR RT primer hybridized to the excess of 3’ SR Adaptor (that which remained free after the 3’ ligation reaction) and transformed the single-stranded DNA adaptor into a double-stranded DNA molecule. dsDNAs were not substrates for ligation mediated by T4 RNA Ligase 1 and therefore did not ligate to the 5’ SR Adaptor in the subsequent ligation step. The 5’ ends adapter was ligated to 5’ ends of miRNAs. Then the first chain of cDNA was synthesized using M-MuLV Reverse Transcriptase (Promega, Madison, WI, USA). Finally, the cDNA was PCR amplified using the 5’-adapter- and 3’-adapter-specific primers. Products were purified in 6% polyacrylamide gel to produce sequencing libraries which were sequenced by Biomarker Technologies (Beijing, China) using the Illumina HiSeq 2000 system (Illumina, Santa Clara, CA, USA).

### 4.3. Sequence Analysis and Identification of miRNAs

The raw reads obtained from sequencing were firstly processed through in-house Perl scripts. In this step, clean reads were obtained by evaluating sequencing quality, calculating the length distribution of small RNA reads, removing low quality reads and adaptor sequences. Subsequently, the clean reads were analyzed by BLAST against the Rfam (ftp://sanger.ac.uk/pub/databases/Rfam/) database, the GenBank noncoding RNA database (http://blast.ncbi.nlm.nih.gov/) and the Repbase database (http://www.girinst.org/repbase/index.html) to exclude rRNA, tRNA, snRNA, snoRNA, other ncRNA sequences and repeat sequences. The remaining reads were used for miRNA identification by comparing them with the mature miRNAs and pre-miRNAs from zebrafish listed in miRbase 21.0, with a maximum of one mismatch. The miRNAs with less than 10 read counts in the three libraries were excluded from further analysis [43]. Given the unavailability of the whole genome information of *M. amblycephala*, the high-quality sequences that were not annotated to the conserved miRNAs were analyzed by BLAST against the zebrafish genome to identify potential novel miRNAs using the miRDeep2 software (V2.0, Biomarker Technologies, Beijing, China).

### 4.4. Analysis of Differentially Expressed miRNAs

To compare the miRNA expressions between the non-infection and infection libraries, the expressions of miRNAs in the three libraries were normalized to obtain the expression in reads per kilobase per million mapped reads (RPKM) using the following formula: Normalized expression = Actual miRNA count/Total count of clean reads ×1,000,000. Then, the differentially expressed miRNAs were identified using the IDEG6 program (http://telethon.bio.unipd.it/bioinfo/IDEG6_form/), which involved the 2 × 2 χ-square test, Fisher exact test, and the Audic-Claverie test with a Bonferroni correction for multiple comparisons and a normalization calculation to adjust for the three libraries. Fold-change ≥1 or ≤−1 and FDR (false discovery rate) <0.01 indicated that the miRNA was differentially expressed.

### 4.5. Prediction and Analysis of miRNA Target Genes

The putative target genes of miRNAs were predicted using the Miranda [69] and RNAhybrid methods [70]. Because of the unavailability of the genomic information of *M. amblycephala*, the sequences of zebrafish genome and EST sequences of *M. amblycephala* in our laboratory were used to predict the potential target genes of miRNAs. Furthermore, to obtain GO annotation of the target genes, the results of BLASTX annotation were analyzed with Blast2GO software and mapped to the categories of GO database [71]. Each of the annotated genes was assigned to detailed GO terms and calculated under the categories of biological process, cellular component, and molecular function by using the online WEGO software [72]. Pathway assignments were generated using the KEGG database [73] and the BLASTX algorithm with an *E*-value threshold of 10^−5^.

### 4.6. RT-PCR and qRT-PCR Analysis of miRNAs and mRNAs

To validate the existence of predicted novel miRNAs, ten novel miRNAs were selected for RT-PCR analysis as described previously [74]. To validate the Illumina sequencing data, nine miRNAs were randomly selected for qRT-PCR analysis. Briefly, approximately 1 μg total RNA was reverse transcribed by using the All-in-One™ miRNA First-Strand cDNA Synthesis Kit (GeneCopoeia, Rockville, MD, USA) following the manufacturer’s instruction. In this kit, poly-A polymerase was used to add poly-A tails to the 3′ end of miRNAs, and M-MLV RTase, with a unique Oligo-dT adaptor primer, was used to reverse transcribe the miRNA tailed poly-A. Forward primers of the selected miRNAs were designed according to the mature sequences of miRNAs (Table S8), and the reverse primer was supplied in the kit. To quantify mRNA expression, approximately 1 μg total RNA was reverse transcribed by using the PrimeScript^®^ RT reagent Kit with gDNA Eraser (TaKaRa, Dalian, China) following the manufacturer’s instruction. Subsequently, qRT-PCR was performed on a LightCycler^®^ 480 II real-time PCR detection system (Roche Diagnostics Deutschland GmbH, Mannheim, Germany) with LightCycler^®^ 480 SYBR Green I Master Mix (Roche Diagnostics) according to the manufacturer’s protocol. qRT-PCR was conducted with the following cycling program: pre-denaturation at 95 °C for 30 s, 40 cycles of 95 °C for 5 s, 58 °C for 20 s, and 72 °C for 20 s. All reactions were performed in triplicate. Relative expression levels of the miRNAs and mRNA were measured in terms of threshold cycle value (*C*t) by the 2^−ΔΔ*C*t^ method [75] using *18S r*RNA as an internal reference [33,76]. We chose *18S r*RNA as internal reference for miRNA expression analysis because our preliminary experiment showed that the expression of this gene was stable after *A. hydrophila* infection *M. amblycephala*. All primers used are listed in Table S8. The expression levels of the miRNAs or mRNAs in the 0 h library were set to 1. The relative expression levels by deep sequencing analysis were presented by 2^log2(treatment/control)^. The expression rates by qRT-PCR among the three libraries were shown by fold change.

### 4.7. Transfections

MAF (*M. amblycephala* fin cells) cells were grown in medium M199 supplemented with 10% fetal bovine serum (FBS; Gibco, Grand Island, NY, USA) at 28 °C [77]. To investigate the effect of miR-375 on the endogenous *TF* and *TFR* mRNA expressions, MAF cells were cultured in six-well plates and transfected separately with miR-375 mimic and mimic negative control (mimic-NC) (GenePharma, Shanghai, China) using Lipofectamine2000 (Invitrogen) according to the manufacturer’s protocol. At 24 h after transfection, the cells were harvested for total RNA isolation to investigate the expression of *TF* and *TFR.* The qRT-PCR was conducted as described in Section 4.6. The miR-375 mimic was synthesized as duplex, and the sequences were: 5′-UUUGUUCGUUCGGCUCGCGUUA-3′, 5′-ACGCGAGCCGAACGAACAAAUU-3′. The mimic-NC (negative control) was similar to miR-375 mimic in composition, but not in specificity: 5′-UUCUCCGAACGUGUCACGUTT-3′, 5′-ACGUGACACGUUCGGAGAATT-3′. Transfection efficiency was assessed by determining the expression levels of miR-375 in MAF cells using qRT-PCR.

### 4.8. Luciferase Reporter Assay

The full 3′ UTR sequences of *TF* and *TFR* were amplified and inserted into the psiCHECK-2 dual-luciferase vector (Promega). Then, the binding sites of miR-375 in the constructed wild-type plasmids were mutated using a point mutation approach as described previously [78]. All the primers used are listed in Table S9. HeLa cells were transfected with 200 ng plasmids (wild-type or mutant) and 25 pmol miR-375 mimic or negative control (mimic-NC) per 24-well using Lipofectamine2000 (Invitrogen) according to the manufacturer’s protocol. Luciferase activity was measured at 24 h after transfection using Dual-Luciferase Reporter Assay System (Promega) according to the manufacturer’s protocol. Relative reporter activities were determined by normalizing Firefly activity to Renilla activity.

### 4.9. Statistical Analysis

Data from qRT-PCR analyses were presented as mean ± SD. One-way ANOVA was performed to examine the differential expressions of miRNAs, *TF* and *TFR* genes after infection with *A. hydrophila*. *p*-Values < 0.05 were considered statistically significant.

## 5. Conclusions

In this study, we identified conserved and putative novel miRNAs and analyzed differential miRNA expression in the liver of *M. amblycephala* infected with *A. hydrophila*. Functional annotation of putative targets of differentially expressed miRNAs indicated potential involvement in the regulation of *M. amblycephala* immune responses to bacterial infection. In particular, we demonstrated that miR-375 targets *TF* and *TFR* and could thus be involved in iron homeostasis, especially during bacterial infection. The novel miRNAs identified in this study can add to the knowledge of miRNA pools of teleost fish, and provide more fundamental information for future studies on miRNAs in fish. Meanwhile, our results provide a meaningful framework for further research on the molecular mechanisms of miRNA-mediated regulation of the interactions between *M. amblycephala* and *A. hydrophila*. Understanding of the involvement of miRNAs in fish immune defenses will provide basic information for the development of effective control strategies against bacterial infections and approaches for breeding disease-resistant fish.

## Figures and Tables

**Table 1:** The top 10 most abundant miRNAs in the small RNA libraries generated from the liver of *M. amblycephala* at various time points post-infection with *A. hydrophila*.

0 h		4 h		24 h	
miRNA	Read Counts	miRNA	Read Counts	miRNA	Read Counts
miR-122	4,185,500	miR-122	1,847,241	miR-122	1,949,028
miR-22a	1,110,016	miR-22a	1,382,260	miR-22a	1,188,866
miR-100	630,796	miR-192	724,173	miR-192	503,300
miR-192	550,271	miR-100	605,151	miR-100	496,321
miR-21	221,724	miR-148	305,995	miR-148	168,563
miR-143	213,361	miR-143	257,189	miR-21	164,521
let-7a	144,256	miR-101a	175,935	miR-126a-5p	143,079
miR-126a-5p	141,629	miR-126a-5p	171,509	miR-143	130,810
let-7e	125,862	let-7e	170,094	miR-146a	106,451
miR-148	107,536	miR-21	144,253	let-7e	105,735

## Supplementary Materials

Supplementary materials can be found at www.mdpi.com/1422-0067/17/12/1972/s1. The data sets supporting the results of this article are available in the National Center for Biotechnology Information (NCBI) repository (SRR2922383).
