# Supplementary material for: Identification and Characterization of MicroRNAs in the Liver of Blunt Snout Bream (Megalobrama amblycephala) Infected by Aeromonas hydrophila
Source: Int J Mol Sci. 2016 Nov 25;17(12):1972. doi: 10.3390/ijms17121972 (PMC5187772; doi:10.3390/ijms17121972)
Supplement: Supplementary file 1 [file ijms-17-01972-s001.zip › ijms-146956-Supplementary Materials/ijms-146956-SI.pdf]

# Supplementary Materials: Identification and Characterization of MicroRNAs in the Liver of Blunt Snout Bream (*Megalobrama amblycephala*) Infected by *Aeromonas hydrophila*

**Lei Cui, Hongtao Hu, Wei Wei, Weimin Wang and Hong Liu**

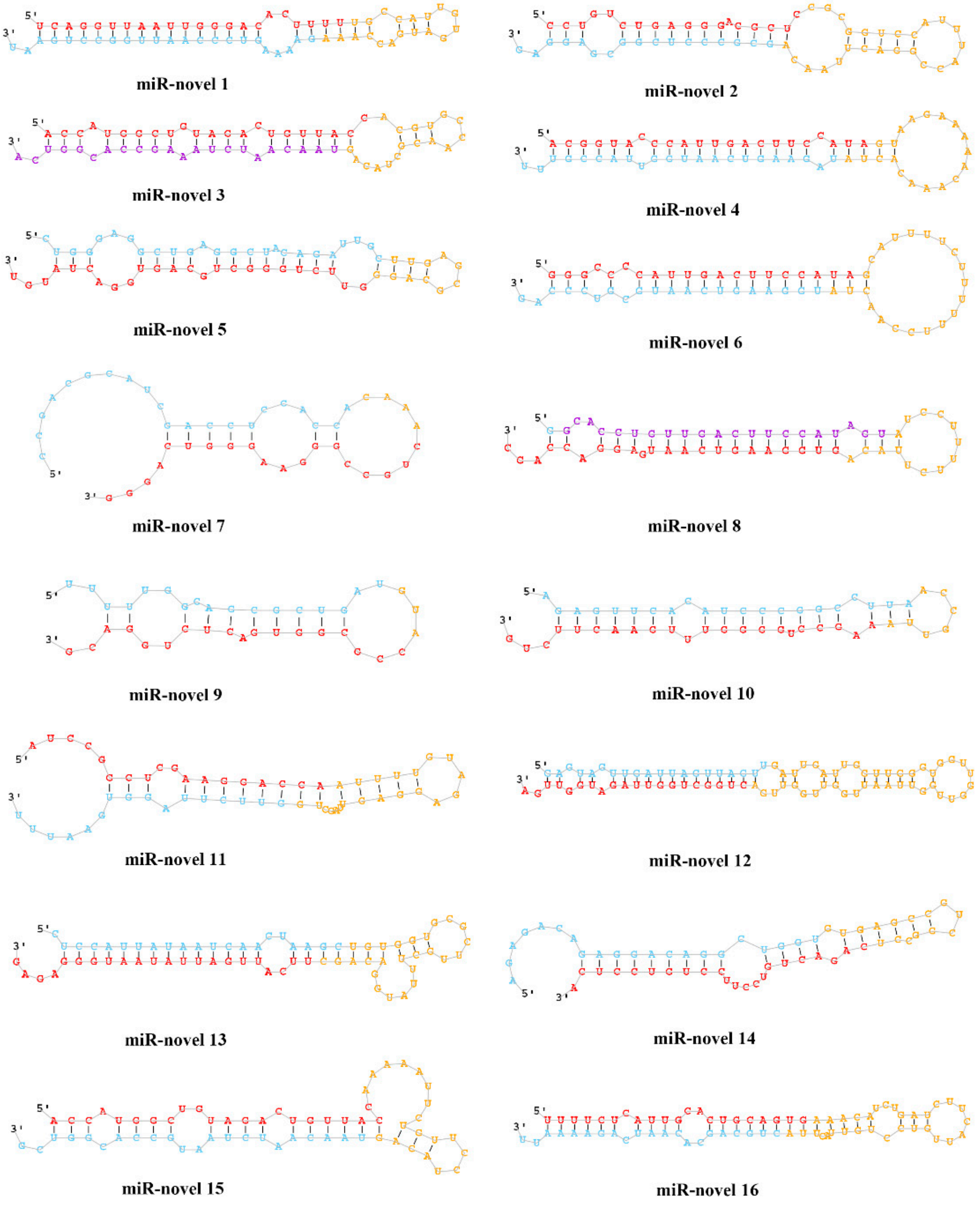


**Figure S1.** *Cont.*

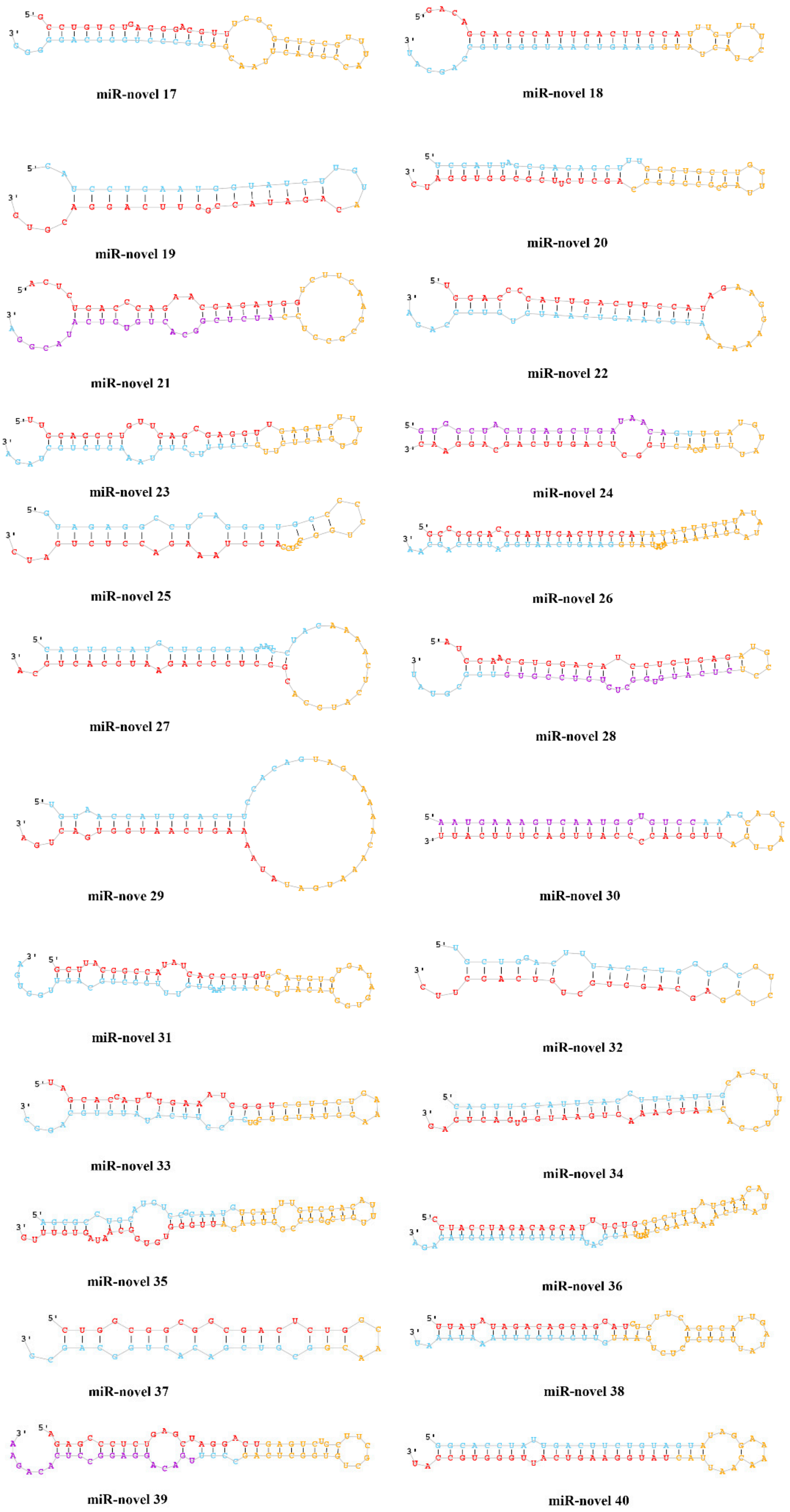


**Figure S1.** *Cont.*

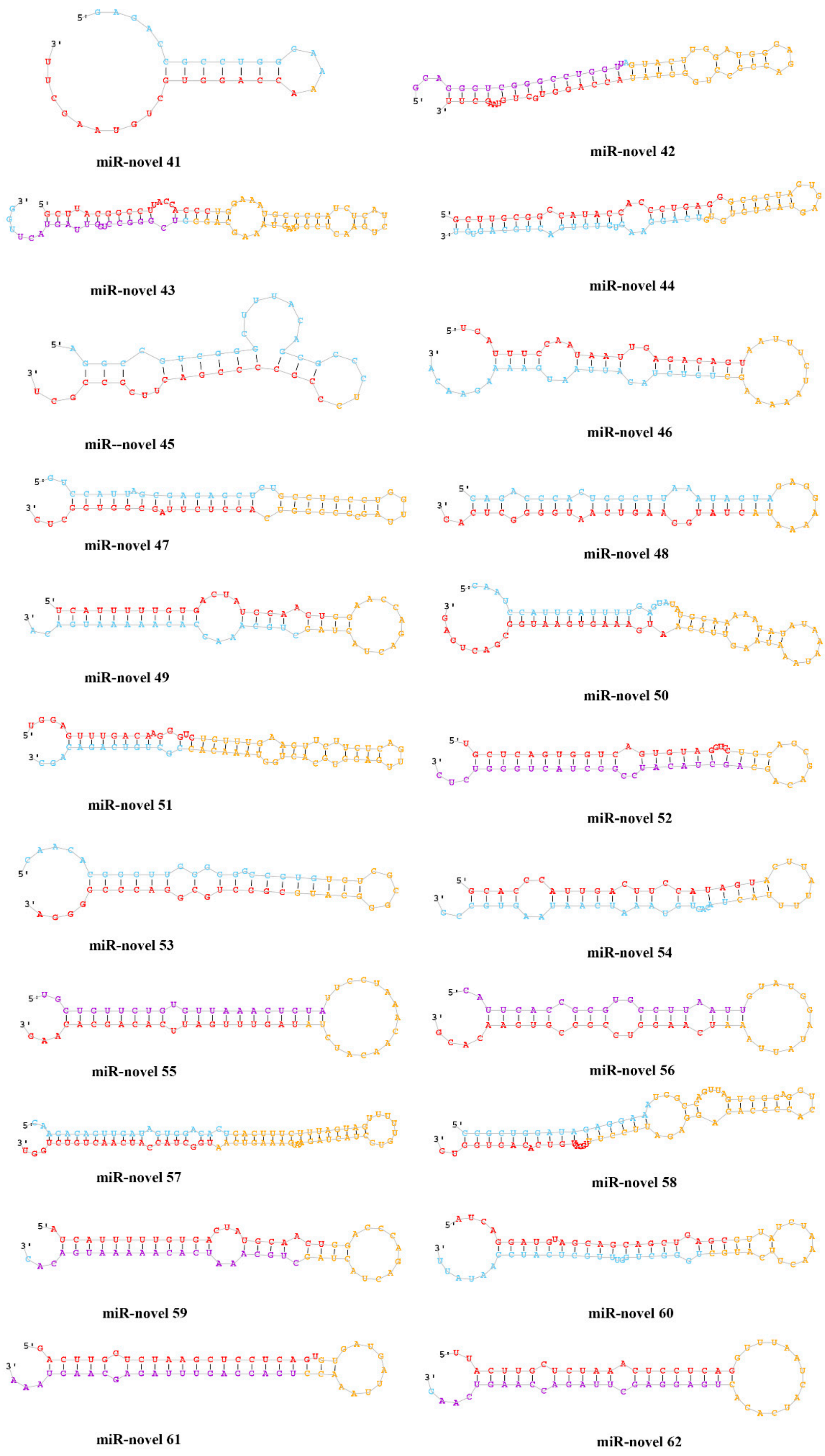


**Figure S1.** Predicted stem-loop secondary structures of all putative novel miRNAs in the liver of *M. amblycephala*.

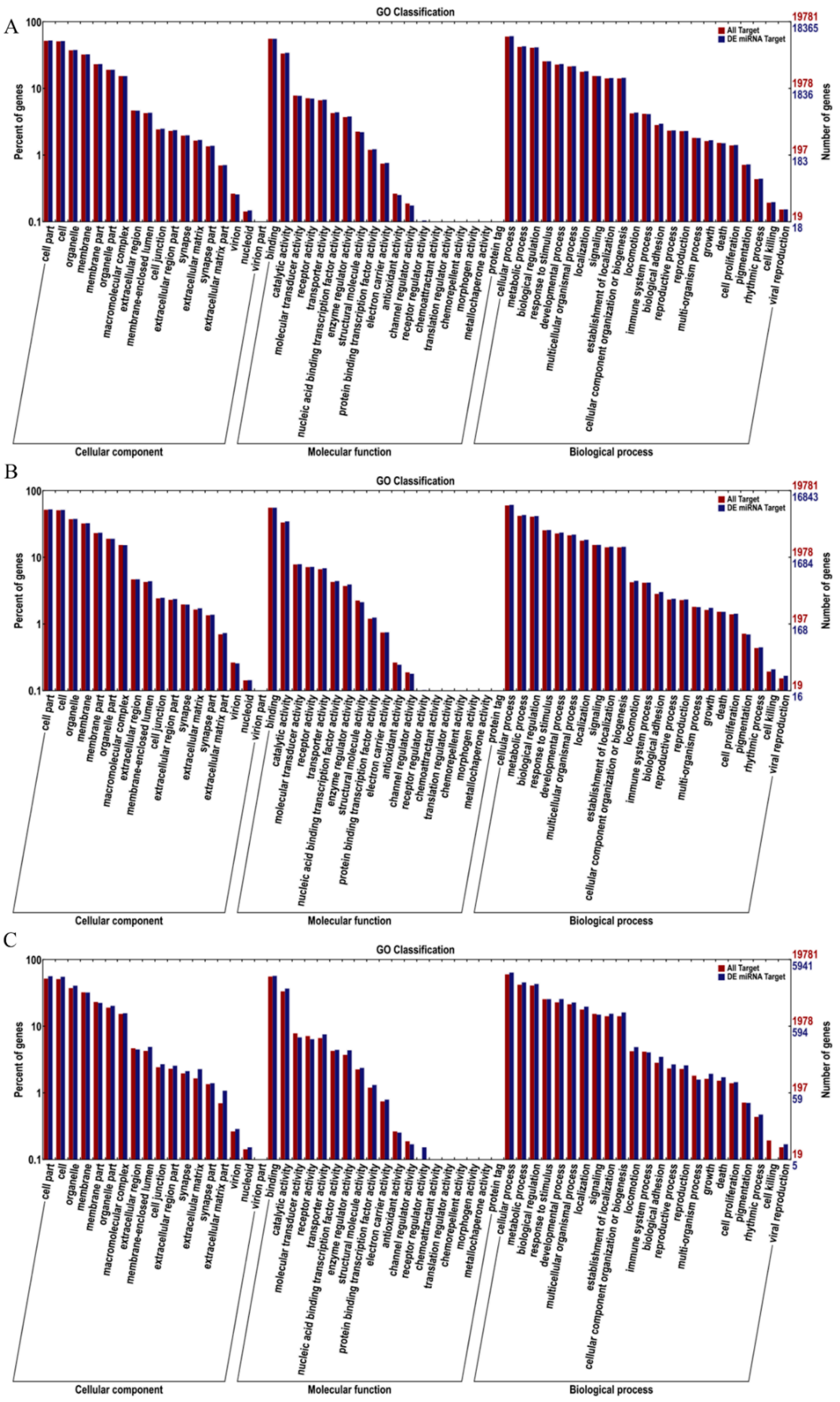


**Figure S2.** GO distribution of target genes of differentially expressed miRNAs in the liver of *M. amblycephala* at various time points post-infection with *A. hydrophila*. (**A**) GO distribution of target genes of differentially expressed miRNAs in the liver of *M. amblycephala* at 4 h post-infection with *A. hydrophila*; (**B**) GO distribution of target genes of differentially expressed miRNAs in the liver of *M. amblycephala* at 24 h post-infection with *A. hydrophila*; (**C**) GO distribution of target genes of differentially expressed miRNAs in the liver of *M. amblycephala* at 24 h post-infection with *A. hydrophila* compared with 4 h.
